# Atypical anti-glomerular basement membrane disease with membranous hyperplasia: diagnostic challenges and treatment variability

**DOI:** 10.1186/s12882-024-03571-5

**Published:** 2024-04-15

**Authors:** Ruoyu Tong, Zhengmao Luo, Xianyang Zhong, Liming Fan, Huangwen Lai, Meng Shen, Yuanhang Huang

**Affiliations:** 1 Nephrology Department, Chinese PLA Southern Theater Command General Hospital, 510010 Guangzhou, China; 2 Pathology Department, Chinese PLA Southern Theater Command General Hospital, 510010 Guangzhou, China

**Keywords:** Atypical anti-glomerular basement membrane disease, Membranous hyperplasia, Kidney

## Abstract

This case report presents a detailed analysis of a 31-year-old male patient who presented with a complex array of clinical symptoms, including proteinuria, hematuria, edema, and kidney insufficiency. Despite undergoing multiple tests, the results for anti-glomerular basement membrane antibodies yielded negative findings. Subsequently, kidney biopsy pathology revealed a distinct diagnosis of atypical anti-glomerular basement membrane (anti-GBM) disease with membrane hyperplasia. Treatment was initiated with a comprehensive approach involving high doses of corticosteroids therapy and cyclophosphamide (CTX). However, contrary to expectations, the patient’s kidney function exhibited rapid deterioration following this therapeutic regimen. The culmination of these complications necessitated a pivotal transition to maintenance hemodialysis. This case underscores the intricate challenges associated with diagnosing and managing rare and atypical presentations of kidney disorders. The negative anti-GBM antibody results and subsequent identification of atypical anti-GBM nephropathy highlight the need for tailored diagnostic strategies to discern subtle nuances within complex clinical scenarios. Additionally, the unexpected response to the treatment regimen emphasizes the potential variability in individual patient responses, underlining the necessity for vigilant monitoring and adaptable treatment strategies. This case report contributes to the evolving understanding of atypical kidney pathologies and the complexities involved in their management.

## Introduction

Anti-glomerular basement membrane disease (anti-GBM disease) is a form of small-vessel vasculitis characterized by the deposition of circulating anti-GBM antibodies within kidney and/or lung tissue. This condition is characterized by the deposition of circulating anti-GBM antibodies within the basement membranes of glomeruli and, at times, the pulmonary alveoli, leading to rapidly progressive glomerulonephritis and, occasionally, pulmonary hemorrhage [1, 2]. However, the clinical and pathological manifestations of anti-GBM disease are not uniform across all cases. Classic anti-GBM disease, often referred to as Goodpasture’s syndrome, primarily involves the kidneys and lungs [3]. The autoimmune response targets the alpha-3 chain of type IV collagen, a major component of glomerular and alveolar basement membranes. The ensuing immune complex deposition triggers a cascade of inflammation, resulting in crescentic glomerulonephritis and, in severe cases, pulmonary hemorrhage [4]. Rapid diagnosis and intervention are essential for favorable outcomes, typically relying on the detection of circulating anti-GBM antibodies and subsequent kidney biopsy to assess disease extent.

Atypical anti-GBM disease is a rare and intriguing kidney disorder that presents with unique clinical and diagnostic challenges [5]. Unlike classical anti-GBM disease, atypical cases deviate from the conventional presentation, making timely diagnosis and effective management paramount [6]. Atypical anti-GBM disease typically exhibits milder clinical manifestations. Firstly, unlike the 34–62% incidence of pulmonary hemorrhage observed in typical anti-GBM disease, atypical cases often lack pulmonary hemorrhage [2, 6, 7]. Notably, Nasr et al. have reported none of the 20 patients with atypical anti-GBM disease presented with pulmonary hemorrhage [8]. Secondly, kidney damage in atypical anti-GBM disease tends to be less severe, primarily characterized by hematuria, proteinuria, and mild kidney insufficiency. This stands in contrast to the acute progressive nephritis syndrome observed in typical anti-GBM disease [5, 6]. The serum creatinine for patients with atypical anti-GBM disease was 2.2 mg/dL, and none of them required dialysis [8]. This suggests that kidney damage is notably milder in comparison to patients with typical anti-GBM disease. Lastly, individuals with atypical anti-GBM disease commonly exhibit negative or low titers of anti-GBM antibodies circulating in their blood, in contrast to patients with typical anti-GBM disease who demonstrate a positive rate ranging from 65–100% [6, 8, 9].

The varying clinical manifestations and atypical presentation can obscure accurate diagnosis, necessitating a comprehensive understanding of the disease’s mechanisms, diagnostic criteria, and treatment approaches. This case report outlines a patient presenting with edema and kidney dysfunction, later diagnosed with atypical anti-GBM disease. As we embark on an in-depth exploration of this enigmatic case, we endeavor to unravel the intricate connections between the diverse clinical features, laboratory findings, and potential underlying pathophysiology. By delving into the complexities of this presentation, we hope to contribute to the evolving understanding of atypical clinical scenarios, thereby enriching the realm of medical knowledge and clinical practice.

## Case presentation

**Fig. 1:**
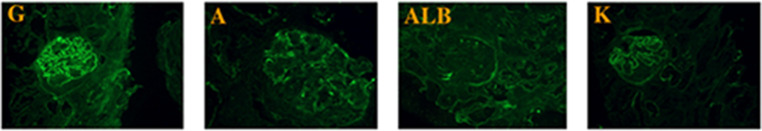
Immunofluorescence analysis of renal biopsy. Immunofluorescence shows diffuse and globular IgG deposits along capillary loops, less pronounced, scattered granular IgA deposits, albumin was barely present within the capillary, and Kappa light-chain deposits. G: IgG, A: IgA, ALB: albumin, K: kappa

A 31-year-old male was admitted to our hospital with facial and double lower limb edema of unknown origin on April 10, 2023. The patient is experiencing symmetrical pitting edema without fever or malaise, and no symptoms of urinary frequency, urgency, or pain. There has been no decrease in urine output or reduction in urine volume. Urinary analysis conducted in the outpatient setting revealed occult blood at 3 + and protein at 2+. After admission, the two-dimensional ultrasound and Doppler flow study of both kidneys revealed no significant abnormalities. The lung CT scan indicated the presence of a small nodule in the anterior segment of the upper lobe of the right lung. As shown in Table 1, the urine routine analysis revealed elevations in erythrocytes (71.2/HPF), leukocytes (5.3/HPF), and urinary protein (4 + g/L). Laboratory results indicated elevated serum levels of creatinine (2.21 mg/dL), cystatin C (3.43 mg/L), uric acid (489 µmol/L), and urea nitrogen (17.8 mmol/L), along with decreased albumin (22.4 g/L). Furthermore, ELISA tests revealed an increased IgG level of 10.90 AU/ml and normal levels of complement C3 (1.2 g/L) and C4 (0.35 g/L). The levels of total anti-β2 glycoprotein 1, anti-protease 3 antibody (C-PR3), and total anti-cardiolipin antibody were also within normal ranges. The preliminary diagnosis included acute kidney failure, nephrotic syndrome, hyperuricemia, and hypertension. Additionally, there is consideration of membranoproliferative glomerulonephritis combined with anti-basement membrane nephropathy, or anti-basement membrane nephropathy. As shown in Fig. 1, kidney biopsy pathology revealed the presence of IgG (++++); IgA (+/-); IgM (+); C3 (negative); C1q (negative); Fib (negative); ALB (negative); kappa (+); and lambda (+). The IgG deposits were found to exhibit a diffuse, globular, and linear pattern along the capillary loops. Anti-glomerular basement membrane antibodies (chemiluminescence) returned negative results on multiple occasions. Additionally, immune and other tests, tumor indicators, as well as infection indicators, all yielded negative results.

**Table 1:** Laboratory test result

Test Item	Result	Reference Range
Urinary Erythrocytes	71.2/HPF	0–2/HPF
Urinary Leukocytes	5.3/HPF	0–5/HPF
Urinary Protein	4 + g/L	-g/L(negative)
Serum Creatinine	2.21 mg/dL	0.74-1.35 mg/dL
Cystatin C	3.43 mg/L	0.51-1.09 mg/L
Uric Acid	489 µmol/L	210–430µmol/L
Urea Nitrogen	17.8 mmol/L	3.1-8.0mmol/L
Albumin	22.4 g/L	40–55 g/L
IgG Level (ELISA)	10.90 g/L	7–16 g/L
Anti-Myeloperoxidase Antibody (P-MPO)	1.81 AU/ml	0-16AU/ml
Total Anti-β2 Glycoprotein 1	< 2.00 AU/ml	0-16AU/ml
Anti-Protease 3 Antibody (C-PR3)	< 2.00 AU/ml	0-16AU/ml
Total Anti-Cardiolipin Antibody	< 5.00 AU/ml	0-16AU/ml
Anti glomerular basement membrane antibody (chemiluminescence)	negative	negative

**Fig. 2:**
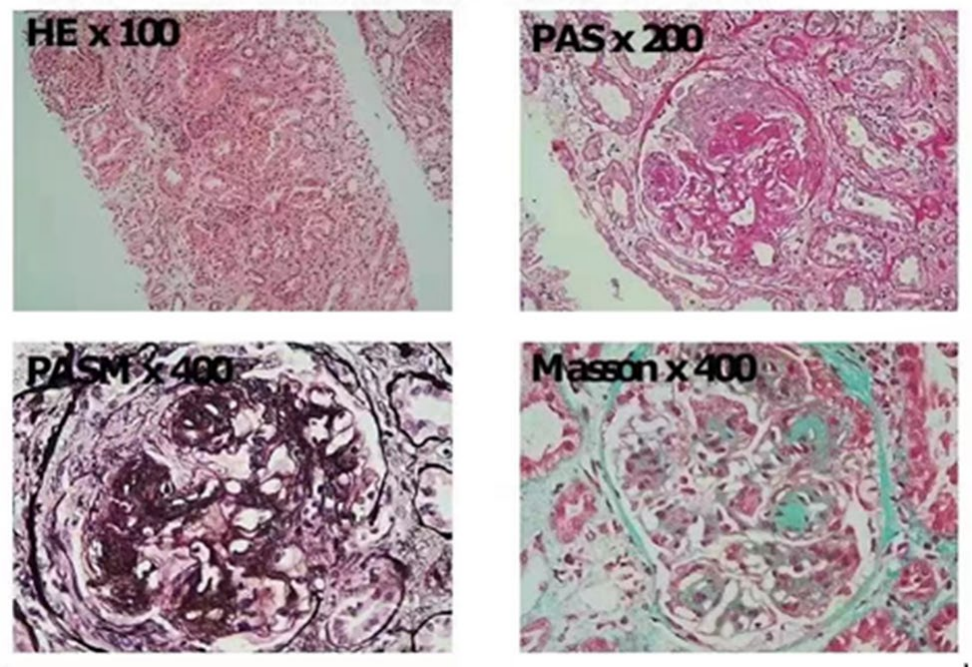
Morphologic analysis of renal biopsy. H&E stain shows general tissue architecture and cell morphology. Periodic Acid-Schiff (PAS) stain highlights basement membranes and mesangial matrix. Jones’ Methenamine Silver (PASM) stain reveals the details of the glomerular basement membranes and mesangial regions. Masson’s Trichrome stain distinguishes between collagen (green) and cellular components (red)

As showed in Fig. 2, we observed 16 glomeruli with no glomerulosclerosis or segmental sclerosis. The glomerular mesangial cells and stroma were moderately to severely hyperplastic with nodular changes, the glomerular capillary loops were lobulated, the capillary lumen was narrowed and occluded, the basement membrane was thickened, and the mesangial insertion and double-track formation could be seen, and there were no angiomatous dilatation and “eyelash-like” structures, no nail-like structures, and no mesangial areas, There was no obvious complex red protein deposition in the mesangial, subepithelial and subendothelial areas. Segmental fibrinoid necrosis was seen in individual glomerular capillary loops, with 2 cellular fibrous, 3 small cellular, and 5 cellular fibrous crescentic formations. Kidney tubular epithelial granular and vacuolar degeneration, a few tubular lumen dilatation, epithelial cell detachment, bristle edge disappearance, multifocal and patchy atrophy (atrophy area of about 55%), kidney interstitium multifocal and patchy inflammatory cell infiltration accompanied by fibrosis, small arteriolar wall thickening, luminal narrowing.

**Fig. 3:**
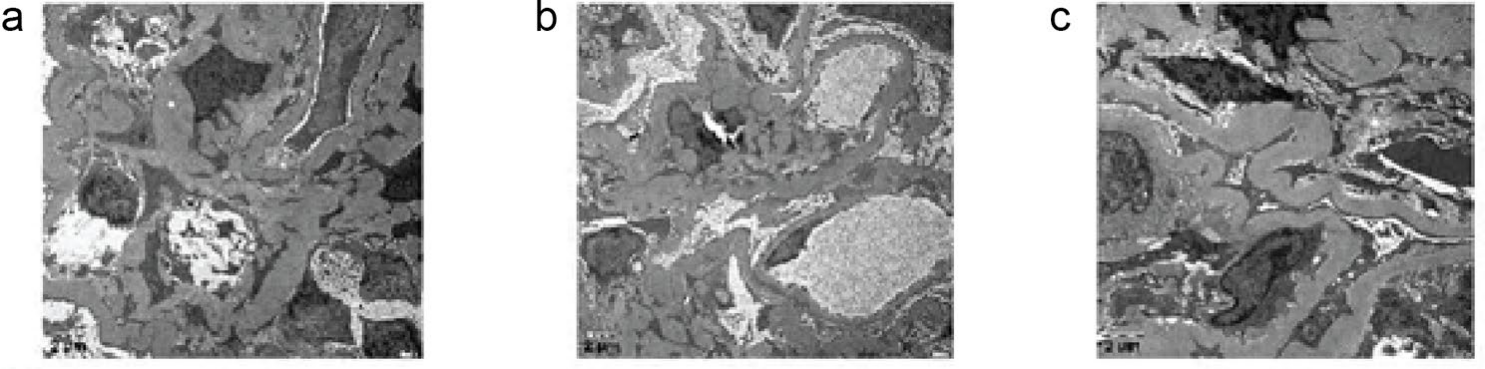
TEM analysis of renal biopsy. (**A**) The left panel shows extensive podocyte effacement and irregular thickening of the glomerular basement membrane (GBM). (**B**) The middle panel reveals subepithelial deposits characteristic of membranous nephropathy, with spikes and dome appearance of the GBM. (**C**) The right panel highlights areas of GBM disruption and cellular interposition, suggesting active glomerular injury. Scale bars represent 2 μm

Electron microscopy analysis in Fig. 3 revealed two glomeruli. One displayed ischemic change with a crumpled appearance, while the other exhibited crescent formation. The latter showed lobulated capillary loops within relatively preserved areas. Marked vacuolar degeneration of endothelial cells, focal endothelial cell hyperplasia, and capillary loop compression were also observed. The mural layer of the kidney capsule exhibited thickening and stratification, alongside mural cell hyperplasia featuring vacuolar degeneration. The basement membrane displayed diffuse, homogeneous thickening up to 1000 nm, with a few tethered insertions. Epithelial cells of the visceral layer exhibited swelling with vacuolar degeneration. The presence of foot process fusion was also noted. Prominent hyperplasia of the mesangial cells was observed, without any electron-dense material deposits. Additionally, vacuolar degeneration of kidney tubular epithelial cells, thickening of the basement membrane in a few tubules, and epithelial cell detachment were noted. The kidney interstitium displayed mild edema along with a minor infiltration of inflammatory cells. Within individual capillary lumens, interstitial erythrocyte aggregates were observed.

The diagnosis was atypical anti-GBM nephropathy with membranous hyperplasia. Methylprednisolone was administered at a dose of 500 mg for 3 days, which was then adjusted to 60 mg/day, in combination with two doses of cyclophosphamide (1.0 g each) administered on May 21 and June 20. The patient commenced dialysis on May 11 due to worsened edema, inadequate diuretic response (furosemide 80 mg, intravenous injection, every other day), and an elevated serum creatinine of 3.47 mg/dL. From July 2 onwards, the patient exhibited symptoms including fever, reduced blood oxygen levels, and tested positive for cytomegalovirus IgM antibodies. A chest CT indicated interstitial pneumonitis. Cytomegalovirus infection was suspected, leading to a reduction in corticosteroid dosage, as well as administration of gamma globulin and ganciclovir as part of anti-viral treatment. The lung infection improved after implementing anti-infection measures. The patient declined further cyclophosphamide treatment. The corticosteroid dosage was reduced to 30 mg/day and combined with 500 mg Mycophenolate Mofetil (MMF) taken twice daily. On July 24th, the patient was discharged from dialysis, and subsequent tests showed blood creatinine levels at 2.28 mg/dL and albumin levels at 26 g/L. After cessation of dialysis, the patient’s corticosteroid dosage was tapered to 15 mg/day of oral prednisone acetate and MMF at a dose of 500 mg twice daily. Diuretic therapy consisted of 20 mg of oral furosemide every other day. One month following this, the patient chose to discontinue steroids and immunosuppressants at an outpatient clinic due to personal preferences.

## Discussion

Anti-GBM diseases are rare spectrum of autoimmune disorders characterized by circulating anti-GBM antibodies that deposit in kidney and/or lung tissues, potentially resulting in kidney damage and/or pulmonary hemorrhage. The target antigen in this context is the non-collagenous domains of the α3(IV)NC1 and α5(IV)NC1 in the GBM [2, 4].

In the development of anti-GBM disease, the immunological characteristics of anti-GBM antibodies play a crucial role in its pathogenicity. This involves a gradual increase in titer, the progressive emergence and amplification of IgG1 subtypes, and a shift in target antigens recognized by the antibodies. Initially limited to α3(IV)NC1 and α5(IV)NC1, the antibodies eventually come to recognize all five target antigens from α1 to α5(IV)NC1 [9, 10]. Plasma exchange has proven effective in removing circulating antibodies, leading to the recovery of pulmonary hemorrhage and kidney function. Recently, there has been the identification of atypical anti-GBM disease, which presents some unique features. Despite revealing typical immunoglobulin deposition along the GBM lineage in kidney immunofluorescence, the serum tests negative for anti-GBM antibodies. Furthermore, the clinical manifestations, serologic and pathologic features, and prognosis of atypical anti-GBM disease differ from those of the typical variant, suggesting the possibility of distinct underlying pathogenic mechanisms [5, 6, 11].

Our patient exhibited pathological features such as diffuse and globular deposition of IgG along capillary loops, diffuse and homogeneously thickened basement membranes, crescent formation (62.5%), moderate-to-severe hyperplasia of mesangial cells, and the visible insertion of mesangial membranes. Additionally, nodular changes were evident in the form of double-track formation. Considering the clinical presentation alongside multiple negative laboratory indicators for anti-GBM antibodies, we concluded that the case involved atypical anti-GBM disease combined with membrane hyperplasia. The absence of electron-dense material in the ultrastructure excluded the possibility of membranoproliferative nephropathy in conjunction with crescent formation.

The linear IgG deposition in atypical anti-GBM disease may be due to an abnormal GBM or non-traditional characteristics of the antibody-binding sites [8, 12]. The phenomenon of “serum negativity” for anti-GBM antibodies could be explained by an “immunological sink,” wherein high-affinity antibodies are selectively sequestered from the circulation, leaving behind low-affinity antibodies undetectable by standard ELISA [13]. This hypothesis is indirectly supported by the fact that when anti-GBM disease was first identified in 1967, it was found that circulating anti-GBM antibodies increased rapidly when patients underwent bilateral nephrectomy [14].

Compared with typical anti-GBM disease, atypical anti-GBM disease develops more slowly. In this case, the patient’s kidney failure progressed slower than typical anti-GBM disease. This was primarily due to acute water and sodium retention from corticosteroids therapy, which was later withdrawn after fluid hemodialysis was restored with oral diuretics. Unlike the clinical presentation of classical anti-GBM disease, it is instead similar to a diabetic nephropathy grade III condition, where the blood creatinine is not high, but the urine output is not at normal levels [15].

Furthermore, the pathology of this case was compounded by membranous hyperplasia. Previous reports have shown that anti-GBM disease can also be associated with or secondary to other glomerulopathies, the most common of which is membranous nephropathy. It is hypothesized that these pre-existing glomerulonephritis may cause damage to the glomerular basement membrane structure, exposing the antigen which is originally in a masked state, inducing autoimmune reaction to produce autoantibodies, leading to the development of anti-GBM disease. In this case, the pathology happened to be combined with a manifestation of membranous hyperplasia as well. Previous reports have shown that anti-GBM disease can also be associated with or secondary to other glomerulopathies, the most common of which is membranous nephropathy. It is hypothesized that these pre-existing glomerulonephritides may cause damage to the glomerular basement membrane structure, exposing the antigen that is originally in a masked state, inducing an autoimmune reaction that produces autoantibodies, ultimately leading to the development of anti-GBM disease. In this case, we favored the pathological manifestation of membranous hyperplasia secondary to atypical anti-GBM nephropathy because no electron-dense material was observed in the ultrastructure, and this case is exceptionally rare. This case was similar to diabetic nephropathy grade III in both clinical presentation and pathology, and it was hypothesized that the two shared a common pathogenesis.

In the management of anti-GBM disease, timely and effective treatment is crucial for improving outcomes. The standard therapeutic strategy includes a combination of immunosuppression (usually with corticosteroids and cyclophosphamide) and plasmapheresis [1, 16]. The goal of immunosuppression is to dampen the immune response and reduce antibody production, while plasmapheresis aims to remove circulating anti-GBM antibodies from the plasma, thereby reducing their pathogenic impact on the kidneys and lungs [17]. For patients with atypical anti-GBM disease, as in our case, treatment decisions can be challenging due to the absence of circulating antibodies detectable by standard assays. However, the presence of organ involvement necessitates a similar approach to that of the typical form. Clinical symptoms and pathology of atypical anti-GBM disease differ from those of typical anti-GBM nephropathy with specificity. An accurate diagnosis based on pathology is essential for the appropriate management of acute and chronic lesions, guiding treatment decisions that may involve massive corticosteroid therapy and cyclophosphamide. Here, we reviewed a total of 20 case reports of pathology of atypical anti-GBM disease. Atypical anti-GBM disease shares similarities with typical anti-GBM disease in linear deposition of IgG. As shown in Table 2, a defining characteristic of atypical anti-GBM disease is negative serum anti-GBM antibodies. Cellular glomerular crescent, a histomorphological indicator of a rupture of glomerular capillaries, is the common pathological finding in both atypical anti-GBM disease and anti-GBM disease. As shows in Tables 2 and 75% (15/20) of atypical anti-GBM disease cases exhibit glomerular crescents. This phenomenon is usually a consequence of an intense immune attack involving cytotoxic elements, such as the complement membrane attack complex and extracellular histones, known as necroinflammation. Mesangial hyperplasia is more common in diabetic nephropathy [18], and it can be observed in 40% (8/20) of cases with atypical anti-GBM disease. If the blood glucose levels and screening of microalbuminuria are normal, it’s not considered diabetic nephropathy [15]. Membranoproliferative glomerulonephritis (MPGN) is identified by specific patterns of glomerular injury seen on a kidney biopsy. These patterns are characterized by distinct light microscopic changes, which include hypercellularity and thickening of the glomerular basement membrane (GBM) [19]. In this case, we observed the thickening of the glomerular basement membrane. However, since we didn’t observe the dense deposits by electron microcopy, MPGN was not considered. Thickening of the glomerular basement membrane is not a common phenomenon in atypical anti-GBM disease, with only 15% (3/20) observed. This highlights the importance of a comprehensive diagnostic approach that considers multiple factors.

**Table 2:** Literature review

Case (age/sex)	Pathology	Immunofluorescence	IgG	anti-GBM antibodies	Electron microcopy
31/M	Proliferative basement membranes, focal crescents, and mesangial proliferation.	IgG (+); IgA (+); IgM (+); C3 (-); C1q (-); Fib (-); ALB (-); kappa (+); and lambda (+).	linear deposition	Negative	No electron-dense deposits and presence of foot process fusion.
57/F [20]	Mesangial proliferation, and focal crescents.	IgG (+); IgA (+); IgM (-); C3 (+); C1q (-); kappa (+); and lambda (+).	linear deposition	Negative	No electron-dense deposits and extensive FPE.
53/M [21]	Proliferative basement membranes, focal crescents, and segmental nodular glomerulosclerosis	IgG1 (+); IgG4 (+).	linear deposition	Negative	Fibrillar deposition.
74/M [22]	Mesangial proliferation, no crescents, and thinning basement membranes	IgG1 (+);IgG4 (+);IgG2 (+); kappa (+); and lambda (+).	linear deposition	Negative	Accumulation of flocculent debris.
43/F [23]	Focal crescents and endocapillary hypercellularity	IgG (+); IgA (-); IgM (-); C3 (-); C4 (-); C1q (-); IgG1 (+);IgG4 (+); IgG2 (-);IgG3 (-); kappa (+); and lambda (+).	linear deposition	Negative	Some FPE
13/F [24]	Mesangial proliferation, focal crescents, some globally or segmentally sclerosed glomeruli	IgG (+); IgA (+); IgM (-); C3 (+);C1q (-); IgG1 (+);IgG4 (+);kappa (+); and lambda (+).	linear deposition	Negative	NA
4/F [25]	Subtle segmental scars, segmental endocapillary hypercellularity, and cellular crescent	IgG (+);IgA (-); IgM (-); C3 (-); C1q (-).	linear deposition	Negative	No electron-dense deposits
30/M [26]	Normal	IgG (+);C3 (+);kappa (+); and lambda (+).	linear deposition	Negative	NA
58/M [27]	Focal crescents and proliferative, and sclerosing glomerulonephritis	IgG (+); IgA (+); kappa (+); and lambda (+).	linear deposition	Negative	NA
79/M [28]	NA	IgG (+); IgA (+); C3 (+); and lambda (+); fibrinogen (+).	linear deposition	Negative	NA
48/F [11]	Some globally sclerotic glomeruli, cellular and fibric crescents, Bowman’s capsules and segmental sclerotic glomeruli, tubular atrophy accompanied by interstitial fibrosis, interstitial infiltration of lymphocytes, monocytes, and plasmocytes	IgG (+)	linear deposition	Negative	No electron-dense deposits and presence of foot process fusion.
37/M [29]	diffuse proliferation in the glomeruli, with increased mesangial proliferation; crescents	IgG (+);C3 (+).	linear deposition	Negative	No electron-dense deposits.
64/M [30]	Necrotizing glomerulonephritis, cellular crescent, and diffuse segmental endocapillary proliferation.	IgG1-κ (+).	linear deposition	Negative	NA
69/M [31]	Mesangial proliferation, diffuse endocapillary hypercellularity, mesangiolysis, and extracapillary proliferation	IgG (+); IgG1 (+); IgG2 (+);IgG3 (-); IgG4 (-);	linear deposition	Negative	No electron-dense deposits, diffuse foot process effacement and subendothelial space widening
70/M [32]	Cellular crescents and necrotic lesions	IgG (+);IgM (-); C3 (-);kappa (+); and lambda (+); fibrinogen (+).	linear deposition	Negative	Proliferative basement membranes
46/F [12]	Cellular crescents and fibrinoid necrosis.	IgG (+); IgG1 (+); IgG2 (+);IgG3 (-); IgG4 (+).	linear deposition	Negative	Normal
53/F [33]	Interstitial fibrosis, tubular atrophy, moderate arteriosclerosis, and moderate arteriolar hyalinosis of moderate chronicity	IgG1-kappa (+); IgG1 (+); IgG2 (+);IgG3 (-); IgG4 (+).	linear deposition	Negative	No electron-dense deposits and extensive FPE
24/M [34]	Cellular crescents and mesangial proliferation,	IgG (+); IgA (+); IgM (-); C3 (-); C1q (-);IgG2 (+); IgG4 (+).	linear deposition	Negative	No electron-dense deposits
33/M [35]	Mesangial proliferation, focal interstitial fibrosis, and tubular atrophy	IgG (+);C3 (+); C4d (-).	linear deposition	Negative	NA
37/M [36]	Cellular crescents	IgG (-); IgA (+).	linear IgA deposition	Negative	NA

+: positive; -: negative. FPE: podocyte foot process effacement. NA: not available

In summary, although atypical anti-GBM disease shares similarities with typical anti-GBM disease in terms of immunofluorescence, where immunoglobulin is deposited linearly along the GBM, its clinicopathologic manifestations, response to treatment, and prognosis differ from those of typical anti-GBM disease. This suggests that it has a pathogenesis distinct from that of typical anti-GBM disease. Therefore, it is recommended to evaluate active and chronic lesions through pathology before deciding whether to pursue treatment involving massive corticosteroid therapy and cyclophosphamide.

## Data Availability

The datasets used and/or analysed during the current study are available from the corresponding author on reasonable request.

## Abbreviations

anti-GBM: anti-glomerular basement membrane
MMF: Mycophenolate Mofetil
CTX: cyclophosphamide
MPGN: Membranoproliferative glomerulonephritis
GBM: glomerular basement membrane
